# Correlation between defect density in mechanically milled graphite and total oxygen content of graphene oxide produced from oxidizing the milled graphite

**DOI:** 10.1038/s41598-018-34109-z

**Published:** 2018-10-25

**Authors:** Zinia Mohanta, Hanudatta S. Atreya, Chandan Srivastava

**Affiliations:** 1 Centre for BioSystems Science and Engineering, Indian Institute of Science, Bengaluru, India; 2 Nuclear Magnetic Resonance Research Centre, Indian Institute of Science, Bengaluru, India; 3 Materials Engineering Department, Indian Institute of Science, Bengaluru, India

## Abstract

It has been reported that defect density in ball-milled graphite lattice increases with the milling time. Guided by this, we hypothesized that the oxygen content of graphene oxide can be substantially enhanced by oxidizing ball-milled graphite and also, the oxygen content would monotonically increase with the milling time as the defect sites would be preferred sites for oxidation. Interestingly, we observed that this correlation was not directly proportional for all milling hours. Even though, the defect density of graphite monotonically increased with milling time, the oxygen content of graphene oxide initially increased and then decreased. This was due to milling time dependent change in the size of the graphite plates and consequent relative abundance of the different oxygen containing functional groups on graphene oxide (GO) produced from the milled graphite.

## Introduction

Graphite oxide (or multi-layered GO) constitutes of hydrophilic graphene sheets bearing oxygen moieties (carbonyl, hydroxyl, epoxy and carboxyl groups) on their basal plane and edges^1,2^. The oxygen content of GO determines its various physicochemical properties namely photoluminescence^3^, adsorption^4^, solubility^5^, zeta potential^6^, electrical conductivity^7^ and thermal conductivity^8^. A lot of focus therefore has been on tuning the degree of oxidation of GO either by modification of the oxidation conditions of graphite or extent of reduction of as-synthesized graphene oxide^9–12^. This report provides a two-step methodology, involving mechanical milling of graphite followed by oxidation of the ballmilled graphite, for synergistically tuning the total oxygen content, relative abundance of different oxygen moieties and size of GO sheets. This holds enormous importance in practical applications of GO. Yeh *et al*.^13^ have demonstrated the performance of GO in photocatalytic water splitting and stated that with the introduction of more oxygen in the GO lattice, the valence band maximum increases as well. Inherently, GO is a p-doped material and its electronic properties can be tailored by tuning its surface functionalities. A beautiful example of effect of size of GO was reported by Zhao *et al*.^14^ GO is the precursor for mass production of graphene. They showed that graphene prepared from large area GO sheets had much lower sheet resistance in comparison to GO prepared from small area GO sheets. Liu *et al*.^15^ compared graphite, graphene oxide, graphite oxide and reduced graphene oxide and observed highest antimicrobial activity by graphene oxide suggesting tailoring of functionalities, size and conductivity can enhance application potential.

A series of GO samples were synthesized using a two-step process. In the first step, planetary ball-milling of graphite for different times was conducted. In the second step, these milled graphitic precursors were oxidized using the Tour’s method^16^ to produce GO. Henceforth, GO synthesized using ‘X’ hours milled graphite is referred as ‘GO_X’.

**Figure 1:**
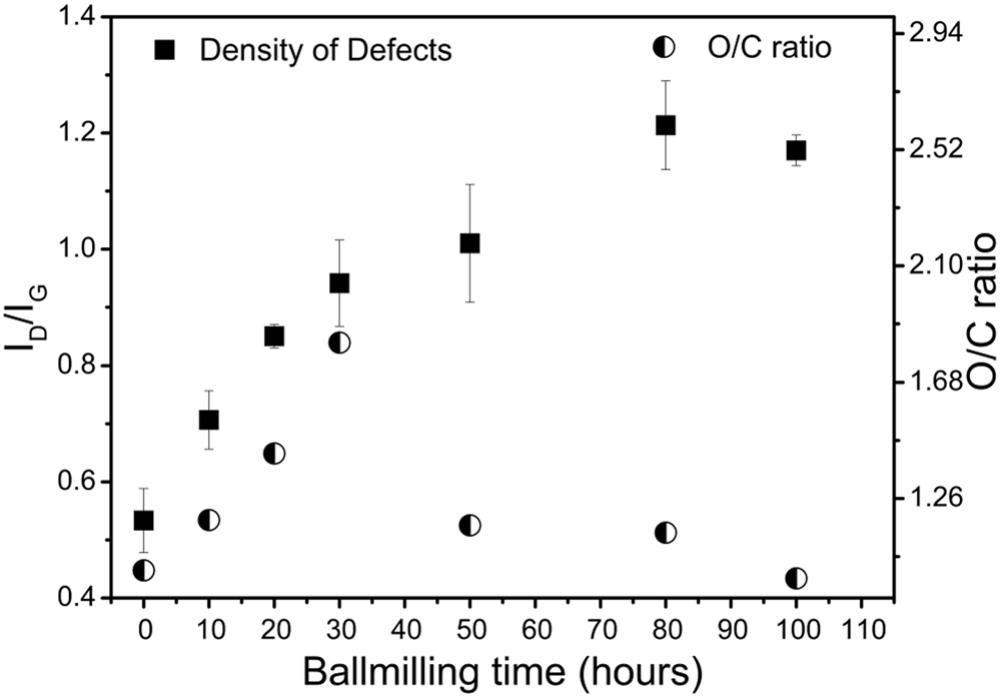
Variation of the density of defects in graphite and oxidation degree in graphene oxide with ball-milling time.

Defect density in the milled graphitic samples was determined from Raman spectrum. The defect density was quantified by the ratio of the intensities of peaks corresponding to the D and G band (Supplementary Table S1) in the Raman signal. With increase in the milling time, a clear increase in the intensity of the D band (I_D_) relative to the intensity of the G band (I_G_) was noticed (Supplementary Fig. S1a). The I_D_/I_G_ ratio (or the defect density) increased gradually from 0.53 at 0 hours to 1.21 at 80 hours followed by a very slight decrease to 1.17 at 100 hours of ball-milling (Fig. 1).

Upon oxidizing the milled graphitic precursors, graphene oxide was obtained as confirmed by the Fourier transform infrared (FTIR) spectra (Supplementary Fig. S2a), solid state nuclear magnetic resonance (ssNMR) spectra (Supplementary Fig. S2a), and X-ray photoelectron spectra (XPS). Thickness of the GO sheets, measured using the atomic force microscopy technique, was in the range of 1–100 nm. This showed the presence of single to multi-layered GO flakes. Oxygen content of GO was determined by the deconvolution of the high resolution C1s scans (Supplementary Fig. S3) obtained from XPS. The oxygen content was quantified by O/C ratio obtained from the relation^17^:

$$\frac{O}{C}ratio=\frac{(2\times {I}_{coo})+(1\times {I}_{C-o})+(1\times {I}_{C=O})}{{I}_{C=C}}$$

where, I_COO_, I_C-O_, I_C=O_, I_C=C_ correspond to the intensities of the peaks corresponding to carboxyl hydroxyl, carbonyl groups and aromatic carbon respectively. Variation of the oxygen content of GO with milling time of graphite precursor is also shown in Fig. 1. As the defects in the graphite lattice increased monotonically with the milling time, we expected a similar trend in the variation of the oxygen content of GO. The oxygen content indeed increased till only 30 hours of milling. Upon further milling, the oxygen content observed was lower than one observed for 30 hours milling. It should however be noted that the oxygen content of the GO produced from the milled graphite sample was always greater than the oxygen content for the GO sample prepared from un-milled graphite.

To understand the above co-relation between defect density and total oxygen content, two different analyses were conducted. Size distribution of the as-synthesized GO was determined using dynamic light scattering experiment and the relative abundance of different oxygen moieties (as determined by deconvolution of the high resolution C1s XPS spectra) from GO samples obtained from graphite milled for different times was compared.

**Figure 2:**
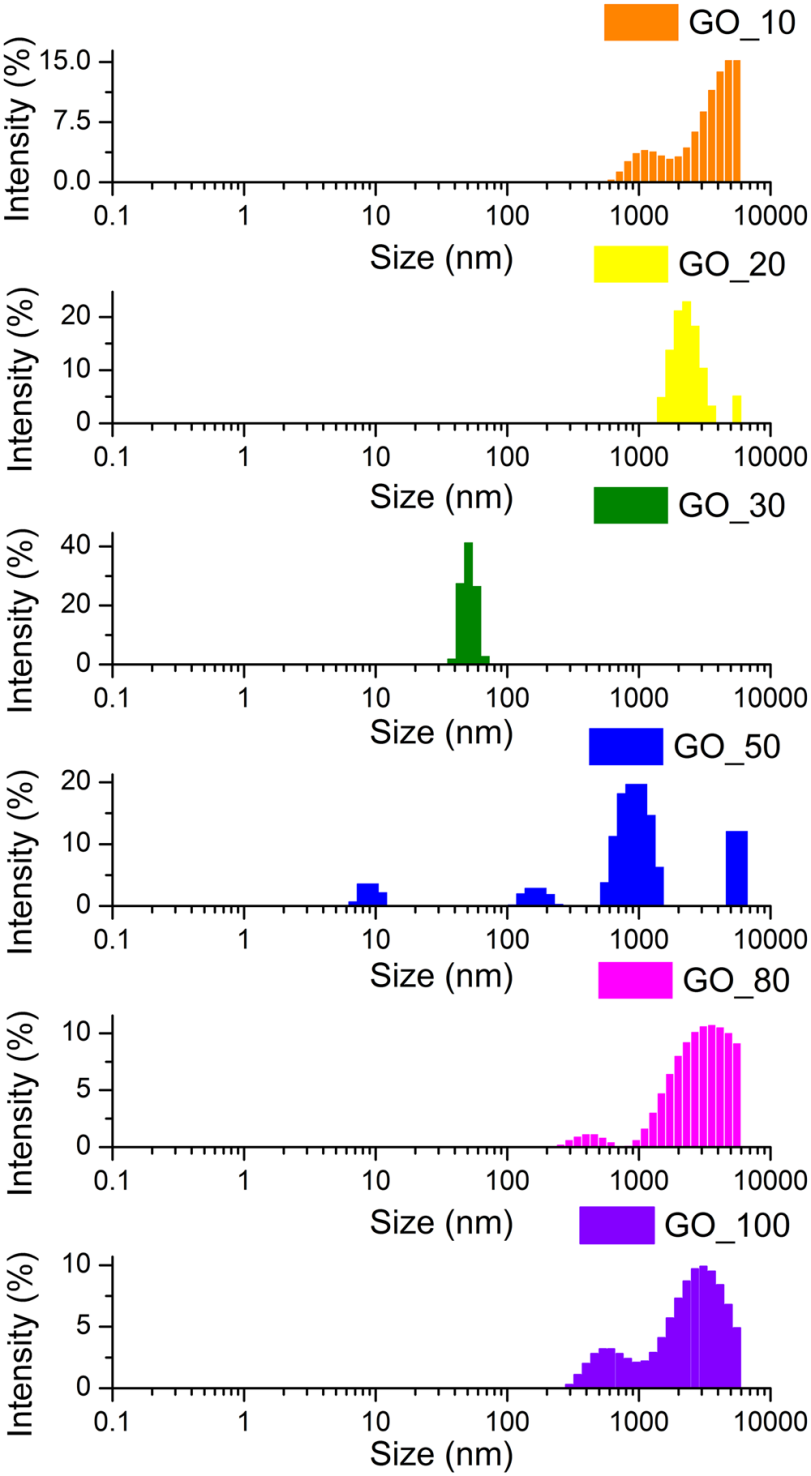
Size distribution of as-synthesized GO produced from graphite ball-milled for different times.

**Figure 3:**
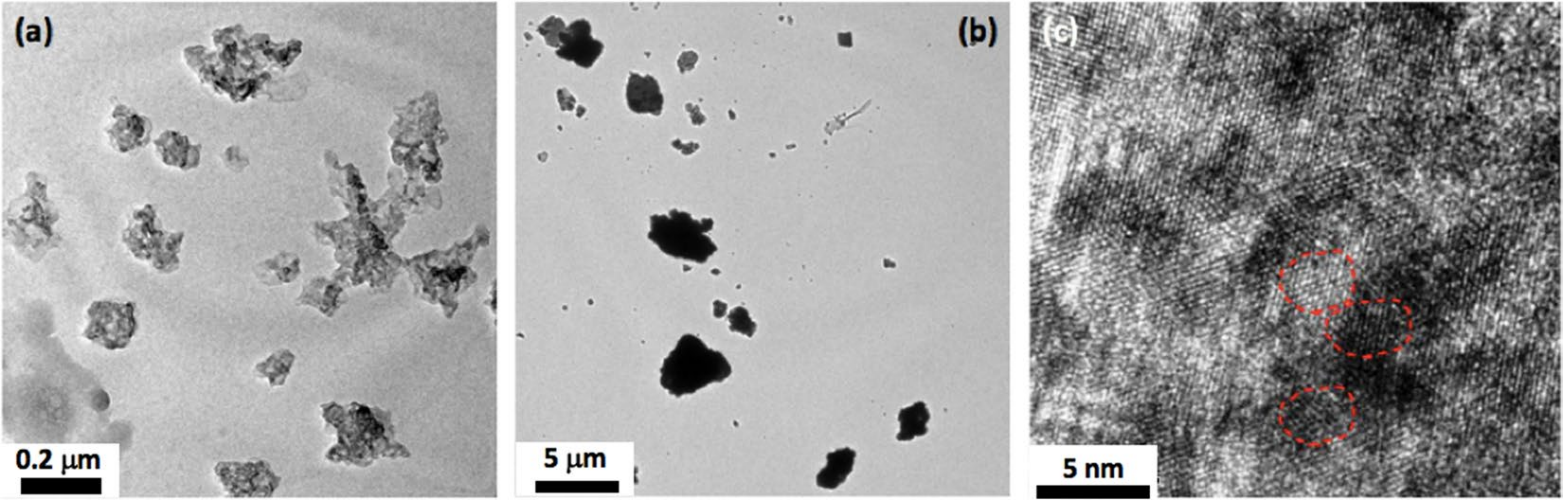
(**a**) Low magnification TEM image of BMG_30, (**b**) Low magnification TEM image of BMG_100, (**c**) High resolution TEM image of BMG_100.

**Figure 4:**
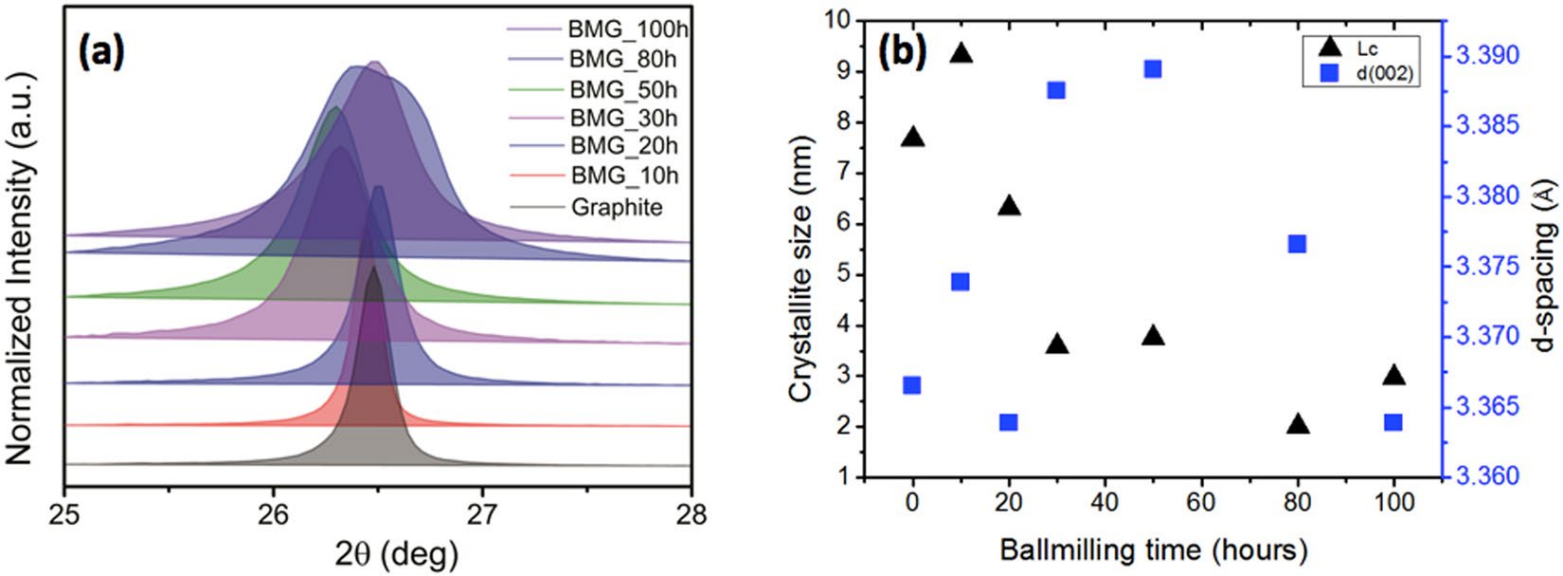
(**a**) XRD curves obtained from ball-milled graphite, (**a**) Variation of grain-size and interplanar spacing in ball-milled graphite particles.

Size distribution histograms for different GO samples are provided in Fig. 2. It can be clearly observed that the size of the as-synthesized GO initially decreased and then increased achieving the minimum size for the GO_30 sample. This indicated towards breaking of the graphitic precursor till 30 hours of milling and then sintering thereafter. The phenomenon involving initial size reduction followed by sintering induced size increase during extended mechanical milling is well known in case of metals. A representative low magnification transmission electron microscopy (TEM) image showing the presence of ~100–200 nm and ~4–5 μm graphite particles in 30 hours and 100 hours milled sample is provided in Fig. 3(a,b) respectively. Sizes of the graphite particles (as seen in the TEM) are in good agreement with the sizes of the GO synthesized from these milled graphite precursors. Breaking of the graphitic plates would increase the edge defects whereas sintering of the graphite flakes (at higher ball milling times) would add in-plane defects due to formation of grain boundaries. This was confirmed by high resolution TEM (HRTEM) imaging for 100 hours milled sample (Fig. 3(c)). The region containing similarly oriented lattice fringes represents grains and the discontinuity of lattice fringes represent grain boundaries. It is clearly observed that 100 hours milled graphitic sample has particles with ultrafine graphitic grains which are randomly oriented. Also, the continued mechanical milling of the graphite would lead to incorporation of defects in the graphite lattice whatever may be their size (Fig. 1). This was also confirmed from the continuous decreases in the Scherrer crystallite size (obtained from the Scherrer formula^18,19^ and FWHM of the (002) peak in the XRD profiles of the milled graphite samples) of the graphite with increase in the milling time. XRD profiles of the milled graphite samples are provided in Fig. 4(a). Variation of the crystallite size and (002) interplanar spacing (d-spacing) of graphite samples as a function of milling time is shown in Fig. 4(b). A negligible change in the interplanar spacing with the milling time illustrates absence of oxidation of graphite during the mechanical milling. The largest observed interplanar spacing for milled graphite was 3.389 Ǻ which is very less the typical interplanar spacing value of ~9.78 Ǻ observed for GO (Supplementary Fig. S2b).

**Figure 5:**
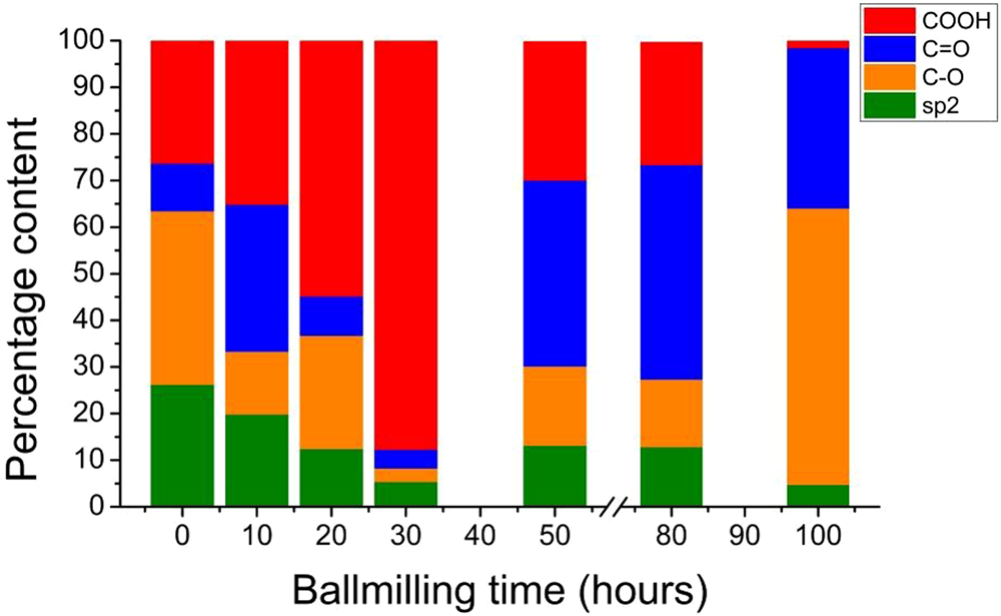
Surface chemistry of GO series. Percentage of aromatic carbon and oxygen-functionalized carbon in graphene oxide with respect to the ballmilling time of respective graphitic precursor, estimated from deconvonlution of XPS spectra.

**Figure 6:**
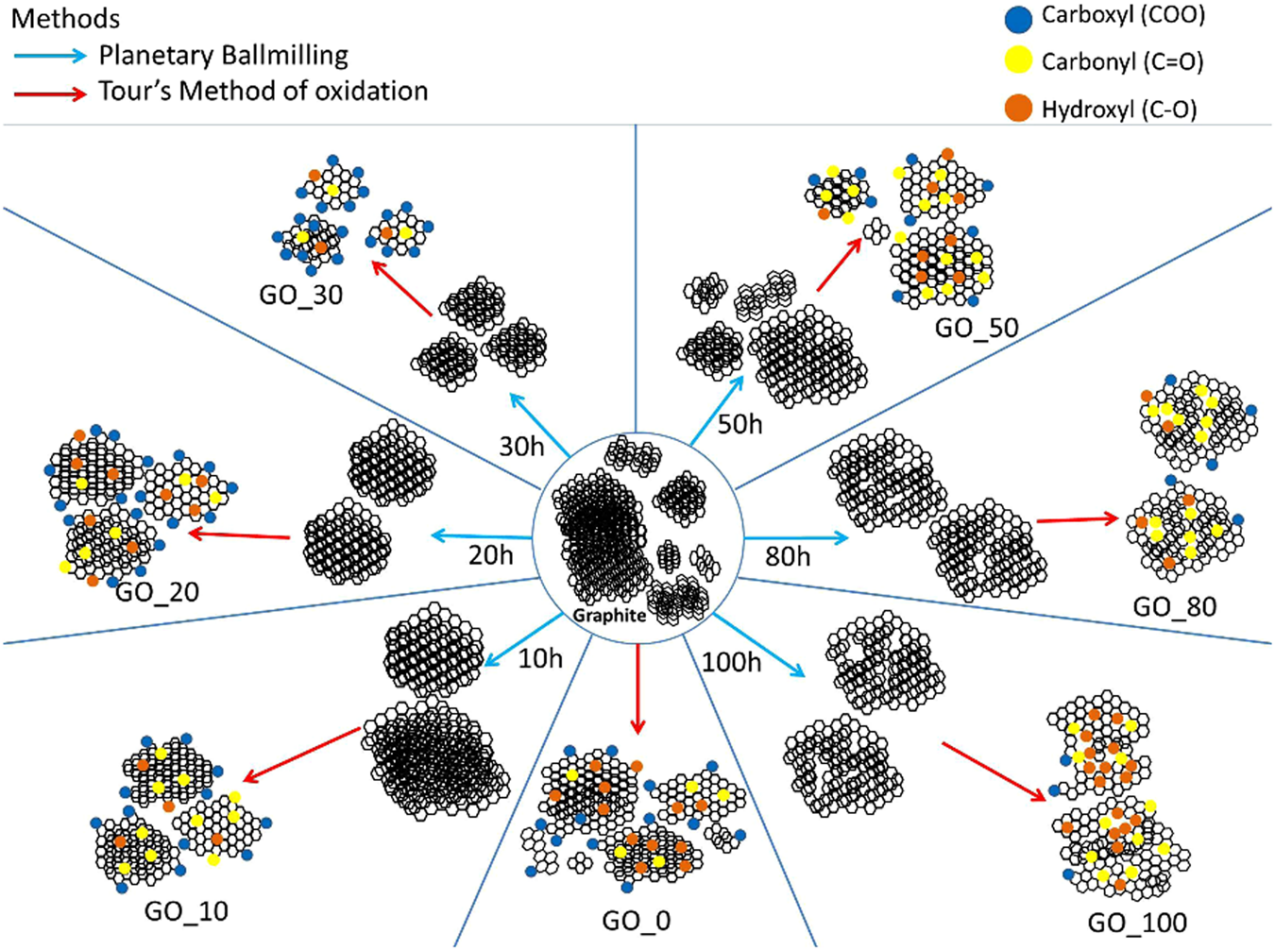
Graphical Summary of GO series prepared through two-step method of ballmilling and oxidation. Graphite is ballmilled for different hours. In the next step, Graphene oxide (GO) is prepared through Tour’s method of oxidation of each graphite (milled and unmilled). Blue arrow represents ballmilling process, red line indicates Tour’s method of oxidation. Green line indicates increase in defects. Circles represent functional groups, blue for carboxyl, yellow for carbonyl, orange for hydroxyl groups. All the structures in this figure are for representation purpose only and are not scalable.

A histogram plot showing the relative abundance of different oxygen containing functional groups (carboxyl, carbonyl and hydroxyl groups) on as-synthesized GO samples is provided in Fig. 5. It can be observed very distinctly that the percentage of carboxyl groups gradually increased till 30 hours of milling followed by a gradual decrease for further milling times. It has been reported that carboxyl group predominantly forms at edges. Therefore, the variation in the relative abundance of the carboxyl groups with milling time is in agreement with size variation of GO flakes with milling time. As among the oxygen moieties attached to the GO, the carboxyl group carries the maximum number of carbon atoms so the total oxygen content of the GO maximizes for the GO_30 sample which contains the maximum amount of edges. A schematic provided in Fig. 6 summarizes this work.

## Methods

The ball-milling setup consisted of two metal grinding jars (rotating about their axis) mounted on a horizontal plate that revolved about the central axis. In ball-milling, the material is pulverized by the friction between the metal balls and the metal bowl. Ratio of weight of graphite to the weight of the stainless steel metal balls was taken as 1:25. The jar was filled with 100 ml of hexane. Bowl speed was set to 300 rpm and the plate speed was set to 720 rpm. The milling was done for 10 minutes followed by a pause of 10 minutes. This was done to avoid excessive heating of the mixture. The temperature of the mixture typically went to ~80–90 C after the milling process. Under such conditions the loss of hexane was minimal. Whatever was the loss was re-filled after 3 cycles.

The ballmilled graphite was then used as precursor for the next step. The ballmilled graphite samples were then oxidized using Tour’s method^16^, with the quantity of each reactant, time of the reaction and frequency of steps optimized for our system. These steps have been elaborated in the Supplementary Informantion.

## Electronic supplementary material

Supplementary information

## Data Availability

The data that support the plots within this paper and other findings of this study are provided in the Supplementary Information and any additional information regarding the data in the paper is available from the corresponding author upon reasonable request.
