# Supplementary material for: Correlation between defect density in mechanically milled graphite and total oxygen content of graphene oxide produced from oxidizing the milled graphite
Source: Sci Rep. 2018 Oct 25;8:15773. doi: 10.1038/s41598-018-34109-z (PMC6202385; doi:10.1038/s41598-018-34109-z)
Supplement: Supplementary file 1 — Supplementary information [file 41598_2018_34109_MOESM1_ESM.docx]

**Correlation between defect density in mechanically milled graphite and total oxygen content of graphene oxide produced from oxidizing the milled graphite.**

**Zinia Mohanta^1^, Hanudatta S Atreya2, Chandan Srivastava3**

^1^Centre for BioSystems Science and Engineering, Indian Institute of Science, Bengaluru, India.

^2^Nuclear Magnetic Resonance Research Centre, Indian Institute of Science, Bengaluru, India.

^3^Materials Engineering Department, Indian Institute of Science, Bengaluru, India

**Supplementary Information**

**Content**

**PS1: Synthesis Step 2 - GO synthesis using Tour’s method**

**PS2: Characterization techniques**

**PS3: Sample Preparation for different studies**

**PS4: Variation in defects of graphite - A Raman Spectroscopy study**

**PS5: Effect of ballmilling on structure on graphite - An XRD perspective**

**PS6: Fourier Transform InfraRed Spectroscopy results - Confirmation of GO signatures**

**PS7: Interlayer spacing of GO - A XRD study**

**PS8: Aromatic and non-aromatic carbon - ssNMR persperstive**

**PS9: Elemental Composition Analysis - XPS study**

**Table S1. Dependence of I_D_/I_G_ (calculated from corresponding Raman spectra) from on milling time**

**Table S2: Relative intensities of the aromatic carbon and non-aromatic carbon deduced from XPS spectra of the GO series**

**Table S3: Physicochemical properties of GO – Summary**

**Fig S1: Characterization of Graphite**

**Fig S2: Characterization of Graphene oxide**

**Fig S3: Deconvolution of XPS High Resolution C1s Spectra of GO**

**Synthesis Step 2 - GO synthesis using Tour’s method**

1 g of graphite and 6 g of KMnO_4_ were mixed in a beaker. 120 ml of sulphuric acid was taken in a beaker and placed in an ice bath. 14 ml of Orthophosphoric acid was added to it to get a 9:1 solution. Both the acids were mixed with glass rod. Then the beaker containing the graphite-KMnO_4_ mixture was kept in the ice bath and the acid solution was poured on the mixture quickly. Adding the solution slowly would result in a highly exothermic reaction. Reaction mixture is stirred with glass rod to get a suspension. The stirring bit was added to and the reaction mixture was placed on a hot plate magnetic stirrer. The reaction mixture was then continuously stirred for 12 hours at 45̊C. Then the reaction mixture is slowly added to another beaker containing 140 ml of DI ice and stirred until the ice melts completely. Then 10 ml of H_2_O_2_ was added to the final mixture. The mixture turned chrome yellow in color. Once the mixture cooled down, 200 ml of DI water was added and was allowed for decantation for 2 days. The sediment was procured and washed using DI water thrice and with ethanol twice. Then it was dried to obtain the GO powder.

**Characterization techniques**

For Raman spectra acquisition, LabRam HR with 532 nm laser was used. The spectra were taken from 1000 to 3000 cm^-1^. To quantitatively study the changes in bands, the bands were fitted with Lorentzian peaks to obtain obtain the peak positions and band intensities in terms of area. JEOL Xpert Pro, PANanalytical JDX-8030 diffractometer (Cu K-alpha radiation, λ=0.1542 nm) was used for XRD (X-Ray Diffraction) analysis. Step size taken was 0.033. Perkin Elmer Frontier FT-NIR/MIR spectrometer was used for the qualitative analysis of the as-synthesized GO samples for authenticating the presence of functional moieties ECXII JEOL 400 MHz with 4mm probe was used for Solid State Nuclear Magnetic Resonance (SSNMR) Spectroscopy. ^13^C Single pulse experiment (delay, d1 of 5 s; number of scans, 8K; spinning speed of 10 kHz) was run for all GO samples. For elemental composition analysis by X-ray Photoelectron Spectroscopy, Kratos XPS Ultra Spectroscope with Al-K-α x-ray source having excitation energy of 1486.6 eV was used. The high resolution C1s spectra were deconvoluted to obtain the relative content of aromatic and nonaromatic carbon in Go and calculate the oxygen content. MALVERN Zetasizer Nano was used for dynamic light scattering (DLS) for obtaining the size distribution profiles and electrophoretic light scattering (ELS) for obtaining zeta potential of the GO series. A 300 keV field emission FEI Tecnai F-30 transmission electron microscope (TEM) was used for obtaining TEM bright field images from as-synthesised samples.

**Fig. S1 – Characterization of Graphite. a)** Raman spectra of the graphite ballmilled with different milling times. An increase in the D band is observed with increase in milling time, indicating increase in defects on crystal lattice.

**a**

**Sample Preparation for different studies**

Dry powder samples were used for Raman Spectroscopy, XRD, ssNMR and FTIR studies. Solution of GO in ethanol were prepared and drop casted on 5*5 mm Si wafers for XPS study. Aqueous solutions of 0.05 mg/ml of GO were prepared for DLS and ELS measurements. Samples for the TEM based analysis were prepared by drop-drying a highly dilute dispersion of the as-synthesised sample onto an electron transparent carbon coated Cu grid.

**Variation in defects of graphite - A Raman Spectroscopy study**

In Raman spectra for Graphite, three distinguishable bands called D, G and 2D bands can be observed at 1350, 1582 and 2718 cm^-1^ respectively (Fig. S1a). The G band corresponds to E_2g_ vibration mode and originates due to the relative motion between a pair of sp^2^ carbon atoms. The D band corresponds to A_1g_ ring breathing mode and arises due to defects present in the lattice. The 2^nd^ order D band, ortherwise known as 2D band arises due to phonon-lattice vibrational process and does not represent defects. The consistent strong 2D band in Fig. S1a denotes that all the samples are multilayered. In pristine graphite, the D band is forbidden, but we observe a D band with low intensity for graphite , as the graphite used is photographic grade. It is clearly evident that the intensity of the D band (I_D_) increases with respect to the G band(I_G_) with increase in ballmilling time till 80 hours and then decreases when ballmilled for 100 hours. The Raman spectra were recorded at 3 different sites for the sample and the relative intensities were calculated for each site for each sample, by integrating the peaks and then obtaining the ratio of the areas of corresponding peaks (I_D_/I_G_). The I_D_/I_G_ ratio was found to increase gradually from 0.53 at 0 hours to 1.21 at 80 hours and then it graphite into smaller crystalline structures and introduction of defects due to distortion of aromatic rings in the graphitic planes. The decrease in this ratio at 100 hours of extended ballmilling may be attributed simultaneous formation of larger clusters due to the welding effect of long ballmilling process.

| Table S1. Dependence of I_D_/I_G_ (calculated from corresponding Raman spectra) from on milling time. I_D_/I_G_ is the ratio of the intensities (integrated area under D and G peaks). Readings are taken at different sites of respective samples. | | | | | |
| --- | --- | --- | --- | --- | --- |
| Ballmilling time (hours) | I_D_/I_G_ | | | Mean | Standard deviation |
| 0 | 0.53 | 0.59 | 0.48 | 0.53 | 0.05 |
| 10 | 0.76 | 0.66 | 0.7 | 0.71 | 0.05 |
| 20 | 0.85 | 0.87 | 0.83 | 0.85 | 0.02 |
| 30 | 0.88 | 1.02 | 0.92 | 0.94 | 0.07 |
| 50 | 0.9 | 1.1 | 1.03 | 1.01 | 0.1 |
| 80 | 1.23 | 1.13 | 1.28 | 1.21 | 0.07 |
| 100 | 1.19 | 1.14 | 1.18 | 1.17 | 0.02 |

**Effect of ballmilling on structure on graphite - An XRD perspective**

All the graphitic samples (unmilled and milled) exhibited single sharp peak in respective XRD pattern. The peak position (2θ) of unmilled graphite was situated at 26.48 deg, which shifted to 26.3 deg for BMG_50h and then shifted to 26.5 for BMG_100h (Fig 4b). In Fig. 4a, it can be noticed that the full width at half maximum (FWHM) and area under the XRD peaks were found to increase with ballmilling time till 80 hours and then decreased for BMG_100. No phase transformations with time could be noticed for the graphite samples as the milling speeds used in this speed are relatively low. The crystallite size along c-axis (Fig. 3d) is calculated using Scherer’s formula.

$$L_{c}=\frac{0.9 \lambda}{\beta\cos\theta}$$

where $\beta$ is the full width at half maximum of XRD peak,$\lambda$ is the x-ray wavelength and $\theta$ is the diffraction angle.

The d-spacing is the spacing between two graphene layers. The interlayer spacing, d is found using the Bragg’s Law,

$$d=\frac{\lambda_{0}}{2 sin \theta}$$

From the information as depicted in Fig. S2b, it was revealed that the d-spacing for graphite in 002 plane increased gradually from 3.36 Ǻ for precursor to 3.39 Ǻ for BMG_50h and decreased to 3.36 Ǻ for BMG_100h. There is very small change in the average interlayer spacing with ballmilling time.

**Fourier Transform InfraRed Spectroscopy results - Confirmation of GO signatures**

In all the FTIR spectra (Fig. S1b)of GO samples, broad and wide peak was observed at 3185 cm^-1^ attributed to O-H stretching vibrations by virtue of C-OH groups and intercalated water. Intense peaks at 1730, 1419 and 1041 cm^-1^ can be attributed to ketone, carboxylic and epoxy groups. The FTIR spectra of the synthesized GO samples exhibit the characteristic peaks of GO for each sample, confirming the presence of oxygen containing functional moieties formed due to oxidation and hence validating successful formation of GO in each case.

**Interlayer spacing of GO - XRD observation**

From XRD information of the GO samples (Fig. 3b), it was revealed that the peak position for GO_0 shifted gradually from 9.51 deg to 9.0 deg for GO_80 and shifted to 9.2 deg for GO_100. Consecutively, the d-spacing of GO_0 increased from 9.3 Ǻ to 9.82 Ǻ for GO_80 and decreased to 9.6 Ǻ for GO_100.

**Aromatic and non-aromatic carbon - ssNMR persperstive**

Characteristic peaks for C=O, COOH, sp2 hybridized carbon, C-O, C-O-C were observed at 190, 168, 132, 70 and 60 ppm respectively in the solid state NMR spectra of all the GO samples(Fig. S2c). In the ssNMR spectrum of GO_0, a sharp peak at 112 ppm is distinguished that can be ascribed to expanded graphite having parent lattice structure. Expanded graphite is the amount of unreacted graphite and does not contribute to graphene oxide. Another significant peak at 20 ppm is detected, which indicates the presence of methyl groups in the samples.

**Fig. S2. Characterization of GO. a)** FTIR spectra of all GO exhibiting characteristic peaks of GO, **b)** Variation of interlayer spacing of GO with respect to ballmilling time of respective graphitic precursor, **c)** ssNMR spectra of all GO. All GO expect GO_0 are completely oxidized. GO_0 shows peak at 110 which is manifested by expanded graphite or unreacted graphite, indicating partial oxidation

**a**

**b**

**c**

**Elemental Composition Analysis - XPS study**

To obtain the nature of carbon in our samples and to determine the contribution of sp2 and oxidized states of carbon, the high resolution C1s scans (Fig. S2d) obtained through XPS were analyzed. Few spectra showing notable hump at 292 eV were considered to have π-π^*^ transitions. π-π^*^ transition (HOMO-LUMO) is characteristic satellite peak arising from carbon from aromatic rings. These spectra were deconvoluted for five components (sp2 at ~285 eV, C-O at ~286.7 eV, C=O at ~287.9 eV, COO at ~290.3 eV and π-π^*^ at ~292 eV) and rest spectra were deconvoluted for four components (sp2 at ~285 eV, C-O at ~286.7 eV, C=O at ~287.9 eV and COO at ~290.3 eV). The contribution of π-π^*^ transitions was neglected. Yet, a very important observation in the XPS spectra was the prominent peaks corresponding to π-π* transitions, which was very large for GO_30. The deconvolution of each high resolution C1s spectrum is demonstrated in Fig. S3. The percentage of the aromatic and non-aromatic carbon (carbon attached to oxygen in any type of bond, namely C-O, C=O, COO here) deduced from these XPS spectra are listed in Table S2.


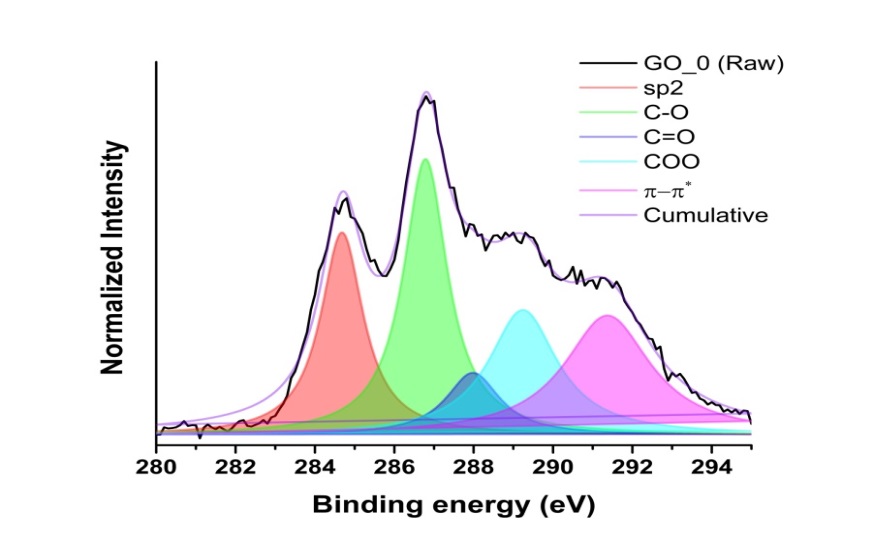

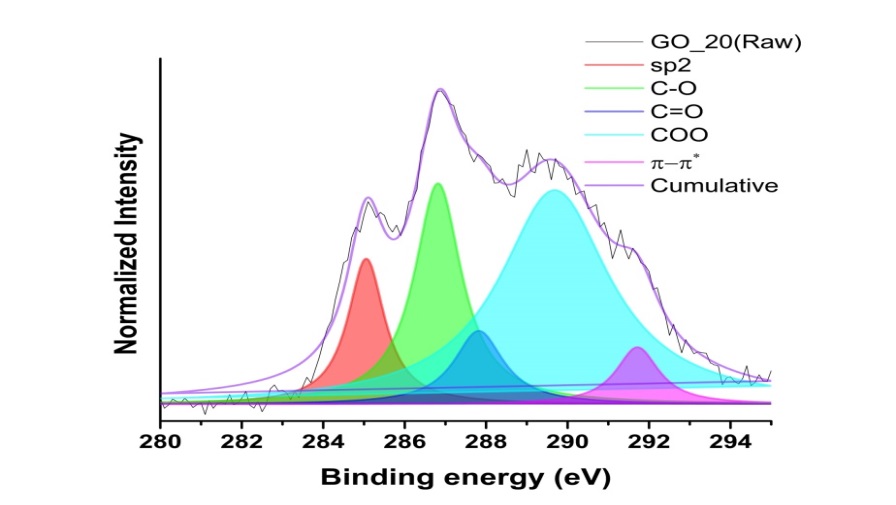

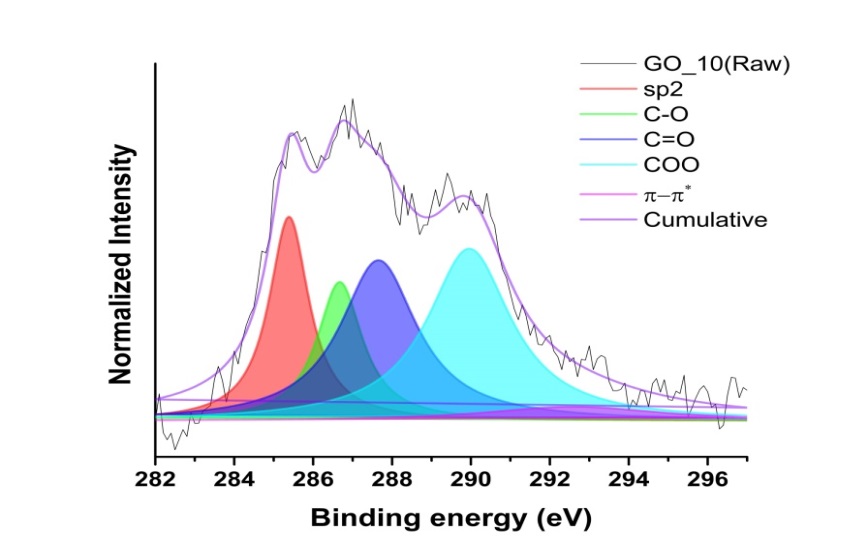

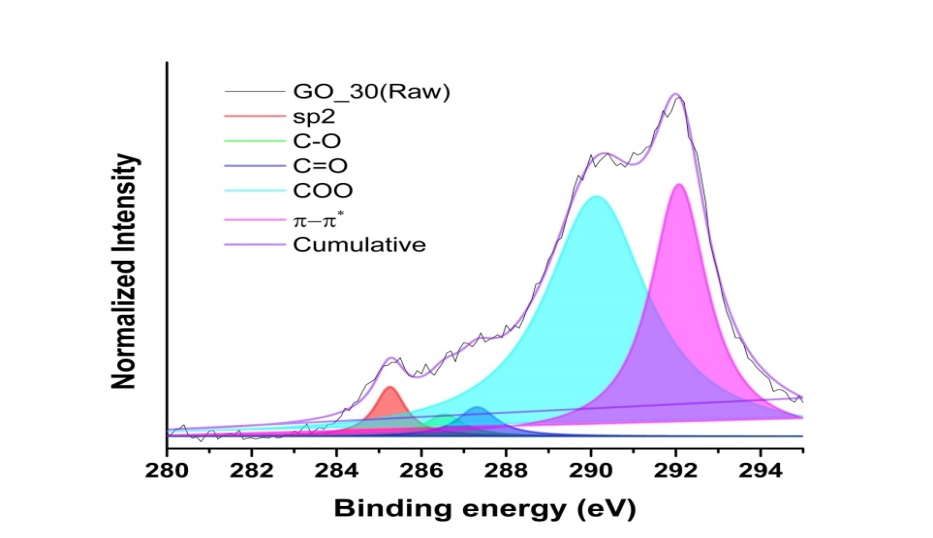

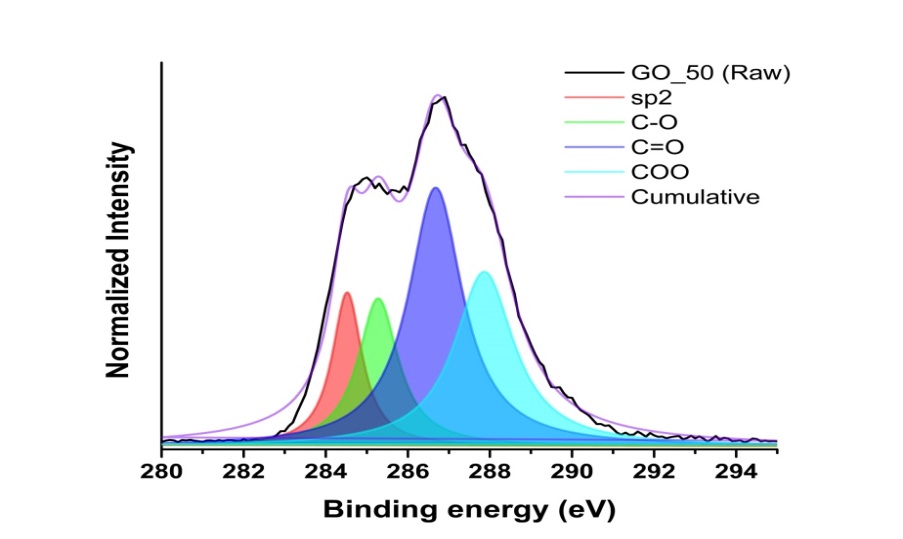

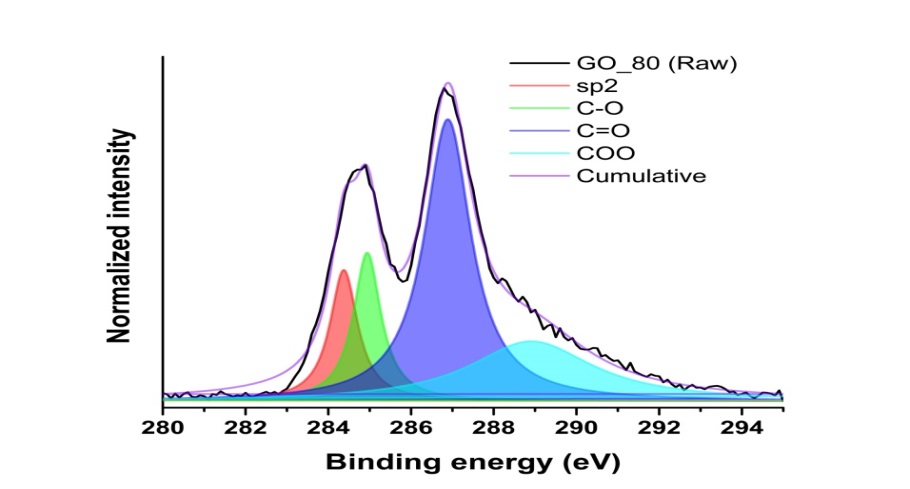


**c**


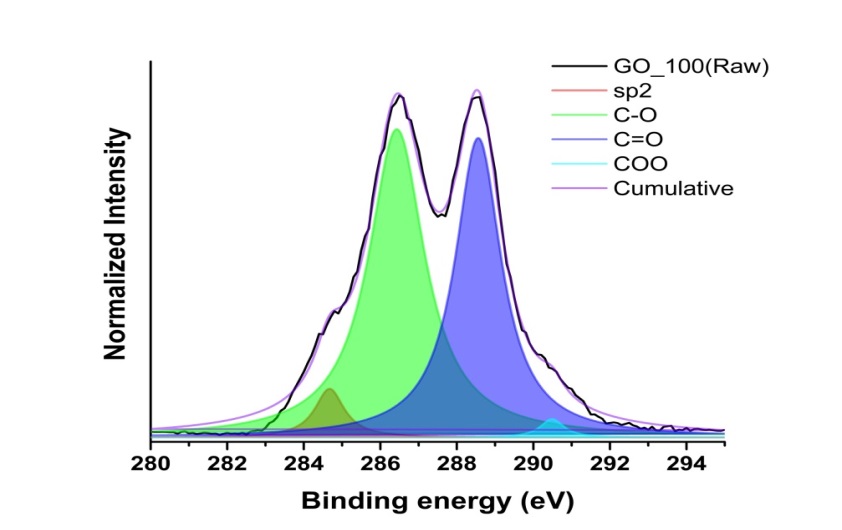


**Fig. S3(a-g). Deconvolution of XPS High Resolution C1s Spectra of GO series. a)** All high resolution C1s spectra of GO were deconvoluted for five components (sp2 at ~285 eV, C-O at ~286.7 eV, C=O at ~287.9 eV, COO at ~290.3 eV and π-π^*^ at ~292 eV), π-π^*^ peak not included for deconvolution where the satellite peak was absent. Intensities (area under corresponding peaks) tabulated in Table S2.

**a**

**b**

**d**

**e**

**f**

**g**

| Table S2: Relative intensities of the aromatic carbon and non-aromatic carbon deduced from XPS spectra of the GO series | | | | |
| --- | --- | --- | --- | --- |
| Sample | C-C | C-O | C=O | COO |
| GO_0 | 26.3 | 37.2 | 10.2 | 26.2 |
| GO_10 | 19.9 | 13.5 | 31.5 | 35.08 |
| GO_20 | 12.5 | 24.3 | 8.4 | 54.68 |
| GO_30 | 5.4 | 2.9 | 4 | 87.58 |
| GO_50 | 13.2 | 17.0 | 39.8 | 29.7 |
| GO_80 | 12.9 | 14.5 | 46 | 26.2 |
| GO_100 | 4.8 | 59.3 | 34.4 | 1.4 |

| Table S3 : Physicochemical parameters of the GO series | | | | | | | |
| --- | --- | --- | --- | --- | --- | --- | --- |
| Sample | GO_0 | GO_10 | GO_20 | GO_30 | GO_50 | GO_80 | GO_100 |
| Interplanar distance in oxidized regions^a^ (Å) | 9.3 | 9.43 | 9.55 | 9.63 | 9.78 | 9.82 | 9.6 |
| Content of non-aromatic carbon^b^ (%) | 73.6 | 80.06 | 87.38 | 94.48 | 86.5 | 86.7 | 95.1 |
| Content of aromatic carbon (%) | 26.3 | 19.9 | 12.5 | 5.44 | 13.2 | 12.9 | 4.8 |
| Content of oxidized GO-type part^c^ (%) | 0.88 | 100 | 100 | 100 | 100 | 100 | 100 |
| C/O ratio | 1 | 0.85 | 0.70 | 0.55 | 0.86 | 0.88 | 1.03 |
| sp_2_/sp_3_ | 0.36 | 0.25 | 0.14 | 0.05 | 0.15 | 0.15 | 0.05 |
| Zeta potential^d^ (mV) | -4.65 | -21.86 | -30 | -34.67 | -24.13 | -28.2 | -34.23 |
| ^a^ Calculated from XRD spectra based on Bragg’s Law  ^b^ Estimated from XPS Spectra  ^c^ Estimated from ssNMR spectra  ^d^ Found through Electrophoretic Light Scattering (ELS). Mean of three readings is tabulated. | | | | | | | |
